# Profiling the orphan enzymes

**DOI:** 10.1186/1745-6150-9-10

**Published:** 2014-06-06

**Authors:** Maria Sorokina, Mark Stam, Claudine Médigue, Olivier Lespinet, David Vallenet

**Affiliations:** 1 Direction des Sciences du Vivant, Commissariat à l’Energie Atomique (CEA), Institut de Génomique, Genoscope, Laboratoire d’Analyses Bioinformatiques pour la Génomique et le Métabolisme, 2 rue Gaston Crémieux, 91057 Evry, France; 2 CNRS-UMR8030, 2 rue Gaston Crémieux, 91057 Evry, France; 3 UEVE, Université d’Evry Val d’Essonne, boulevard François Mitterrand, 91057 Evry, France; 4 Univ Paris-Sud, Institut de Génétique et Microbiologie, UMR8621, Orsay F-91405, France; 5 Univ Paris-Sud, Laboratoire de Recherche en Informatique, UMR8623, Orsay F-91405, France; 6 CNRS, Orsay F-91405, France

**Keywords:** Orphan enzyme activities, Enzyme discovery, Metabolic pathways, Enzyme promiscuity, Data survey, Biological databases, Local orphan enzymes

## Abstract

The emergence of Next Generation Sequencing generates an incredible amount of sequence and great potential for new enzyme discovery. Despite this huge amount of data and the profusion of bioinformatic methods for function prediction, a large part of known enzyme activities is still lacking an associated protein sequence. These particular activities are called “orphan enzymes”. The present review proposes an update of previous surveys on orphan enzymes by mining the current content of public databases. While the percentage of orphan enzyme activities has decreased from 38% to 22% in ten years, there are still more than 1,000 orphans among the 5,000 entries of the Enzyme Commission (EC) classification. Taking into account all the reactions present in metabolic databases, this proportion dramatically increases to reach nearly 50% of orphans and many of them are not associated to a known pathway. We extended our survey to “local orphan enzymes” that are activities which have no representative sequence in a given clade, but have at least one in organisms belonging to other clades. We observe an important bias in Archaea and find that in general more than 30% of the EC activities have incomplete sequence information in at least one superkingdom. To estimate if candidate proteins for local orphans could be retrieved by homology search, we applied a simple strategy based on the PRIAM software and noticed that candidates may be proposed for an important fraction of local orphan enzymes. Finally, by studying relation between protein domains and catalyzed activities, it appears that newly discovered enzymes are mostly associated with already known enzyme domains. Thus, the exploration of the promiscuity and the multifunctional aspect of known enzyme families may solve part of the orphan enzyme issue. We conclude this review with a presentation of recent initiatives in finding proteins for orphan enzymes and in extending the enzyme world by the discovery of new activities.

**Reviewers:**

This article was reviewed by Michael Galperin, Daniel Haft and Daniel Kahn.

## Review

New progress in sequencing technologies generates thousands of new sequences each day. With the large public sequence databases combined with efficient bioinformatic methods, it is possible to predict the function of some new proteins mainly by comparative genomics approaches. Nevertheless, millions of protein entries are not assigned reliable functions due to the lack of trustworthy annotations and the drawbacks of homology-based predictions [1]. This shortcoming illustrates our limited knowledge of the functional diversity in the protein world and restricts the analyses of an organism starting from its genome. This is particularly the case for enzymatic activities that can be predicted by gene functional assignments and used as a starting point to reconstruct genome-scale metabolic models.

The first enzyme was discovered and isolated in 1833 by Anselme Payen [2]. It was the first time a non-living compound was shown to have properties of an organic catalyst, a discovery which shook the scientific community. This enzyme was named “diastase” (now called α-amylase) and the suffix –‘ase’ will be henceforth used to refer to enzymes. Since then, the number of discovered enzymes has continually increased, thanks to the experimental work of chemists and biologists. In the beginning of enzymology, the naming of enzyme was not systematic. Many different enzymes were given similar names and, on the other hand, the same enzymes had several names. An Enzyme Commission, whose first meeting took place in 1961, was created to give rules and recommendations that could be implemented for the systematic naming of enzymes [3]. Enzyme activities are nowadays classified with EC (Enzyme Commission) numbers, a nomenclature maintained by the IUBMB (International Union of Biochemistry and Molecular Biology) [4-6]. To be integrated into the EC classification, an activity must be observed and biochemically characterized without the necessity to identify the associated protein that catalyzes the reaction.

**Figure 1:**
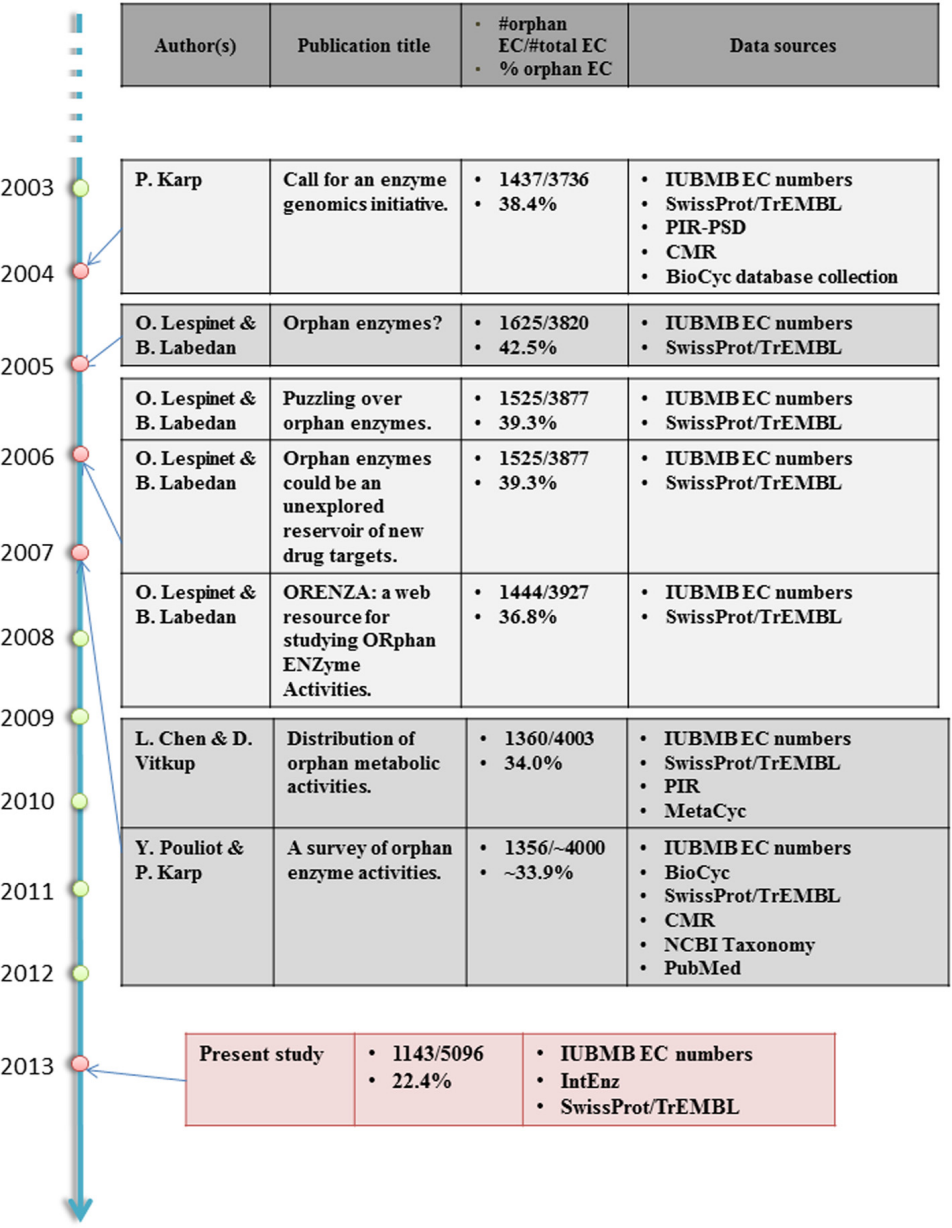
**Orphan enzyme chronicles.** Studies on orphan enzymatic activities in the past ten years.

Since 2003, several teams around the world have noticed that many EC numbers have no identified coding sequences for the enzymes catalyzing the corresponding activities (Figure 1). In order to fill the missing knowledge between genes and their function, Richard J. Roberts called, in 2004, for a community action for the annotation of genes of unknown function in microbial genomes [7]. The same year, Peter Karp proposed an enzyme genomic initiative to associate at least one protein sequence for every biochemically characterized enzymatic activity [8]. He noticed that many EC numbers (38% among 3,736 entries) were lacking an associated nucleic or protein sequence in public databases, a problem that hadn’t been really considered before by the scientific community. He observed that his estimation could be biased as the EC classification does not cover all known enzymatic activities. Indeed, in sequence databases, some entries are missing an EC number even if a correct textual description of the enzymatic activity is annotated. He proposed to take advantage of the numerous accessible sequenced genomes and to cross this genetic information with published experiments that have characterized the enzymatic activities. This first data mining step should identify some candidate proteins which could be experimentally validated.

In 2005, sequence-lacking enzymatic activities were named “orphan enzymes” by Bernard Labedan and Olivier Lespinet in an open letter [9]. They conducted a similar analysis to that of Peter Karp and showed that 42% of the EC numbers were orphan enzymes (1,625 EC numbers among 3,820). One of the main surprises of this study was the fact that 200 organisms had orphan enzymes, despite the availability of their complete genome. They also noticed that, in several cases, the protein catalyzing the enzymatic activity had been identified but not sequenced. The following year they published two exploratory articles on orphan enzymes [10,11]. The proportion of orphan enzymes was updated, giving a slight decrease of 3% (39% of orphans, 1,525 EC entries among 3,877). They pointed out that a number of pathways (~100) had at least one orphan enzyme. They also made several remarks on the use of EC numbers. Moreover, they created a public database, called ORENZA, listing all orphan enzymes present in the EC nomenclature and allowing users to perform queries by tracking them between organisms and pathways [10].

In 2007, Lifeng Chen and Dennis Vitkup carried out a very detailed review on the historical accumulation of orphan enzyme activities and a wide range of statistical analyses on their distribution across different classifications [12]. They found 1,360 orphans, representing 34% of the 4,003 valid EC entries. They investigated the number of biochemical characterizations per year of discovery and noticed that it decreased in the 1970s and 1990s. A study of the relation between orphan enzymes and their pathway neighbors was conducted: 39% of network neighbors for orphan activities were orphan themselves, compared with 29% for neighbors of non-orphan activities. They also noticed that a majority of orphan activities were found in the most studied organisms. Finally, they pinpointed a possible bias in the EC classification because many reactions in metabolic databases were not associated with any EC number. Considering this limitation, they estimated that up to 50% of all know biochemical reactions were orphan.

Here, we present an extended review on orphan enzyme activities by updating previously conducted surveys and performing new analyses. We first update the estimation of the number of orphan enzymes and interpret their decrease in the light of past and recent enzyme activity discoveries. As the EC classification does not totally cover all known activities, we briefly introduce two main metabolic databases and analyze their content to estimate orphans at the reaction level. Also, an analysis of their connectivity in metabolic network is made. The concept of orphan enzymes is then extended to local orphans (*i.e.* activities which have no representative sequence in a given clade, but have one in other organisms) and an analysis is made at the superkingdom level to estimate their number and to evaluate if candidate proteins for local orphans could be retrieved by sequence homology. Finally, we expose the notion of promiscuity and multifunctionality in the enzyme world and explore the relation between protein domains and catalyzed activities. In conclusion, we present some new initiatives and concepts of interest to reduce the number of orphan enzymes but, also, to extend the landscape of enzymes by finding new activities.

### An updated view of orphan enzymes

In this study, we estimated the number of orphan enzymes by using EC numbers present in the IntEnz [13] and UniProt [14] databases (versions of February 2013). UniProt is a resource of proteins where enzymatic activities are described using the EC classification. Only valid and complete EC entries were considered without taking into account deleted or transferred entries. We also considered as valid entries the nearly 100 provisional EC numbers of IntEnz waiting to be confirmed by the IUBMB. It appears that 22.4% of the enzymatic activities are orphans; among the 5,096 EC numbers, 1,143 entries have no associated protein in UniProt. As noticed previously [12], the proportion of orphan enzymes is not uniformly distributed across the different classes of the EC nomenclature: in EC class 1 the fraction is 25%, 26% in class 2, 19% in class 3 and 4, 15% in class 5 and 13% in class 6 (Additional file 1: Figure S1.1 and Additional file 2: Table S2.1 for the complete list of orphan EC numbers).

**Figure 2:**
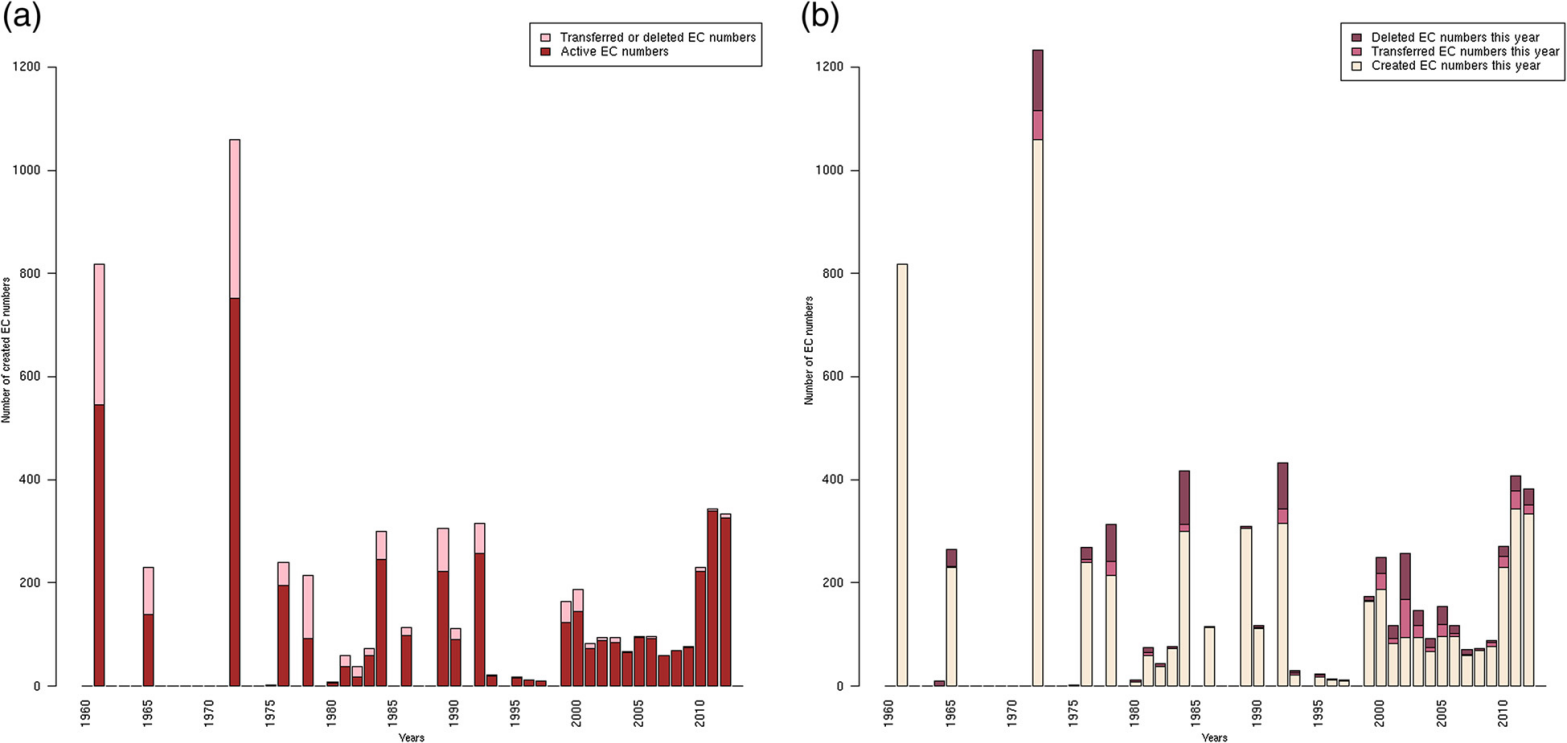
**EC classification evolution over years. (a)** Snapshot of EC number status by year of creation. This barplot represents the number of created EC numbers over years and the proportion of nowadays active entries in red and transferred/deleted entries in pink. **(b)** Dynamics of the EC entry creations and status changes over years. This barplot represents the number of EC entry modifications over years: creation (yellow), transfer (light red) and deletion (dark red).

**Figure 3:**
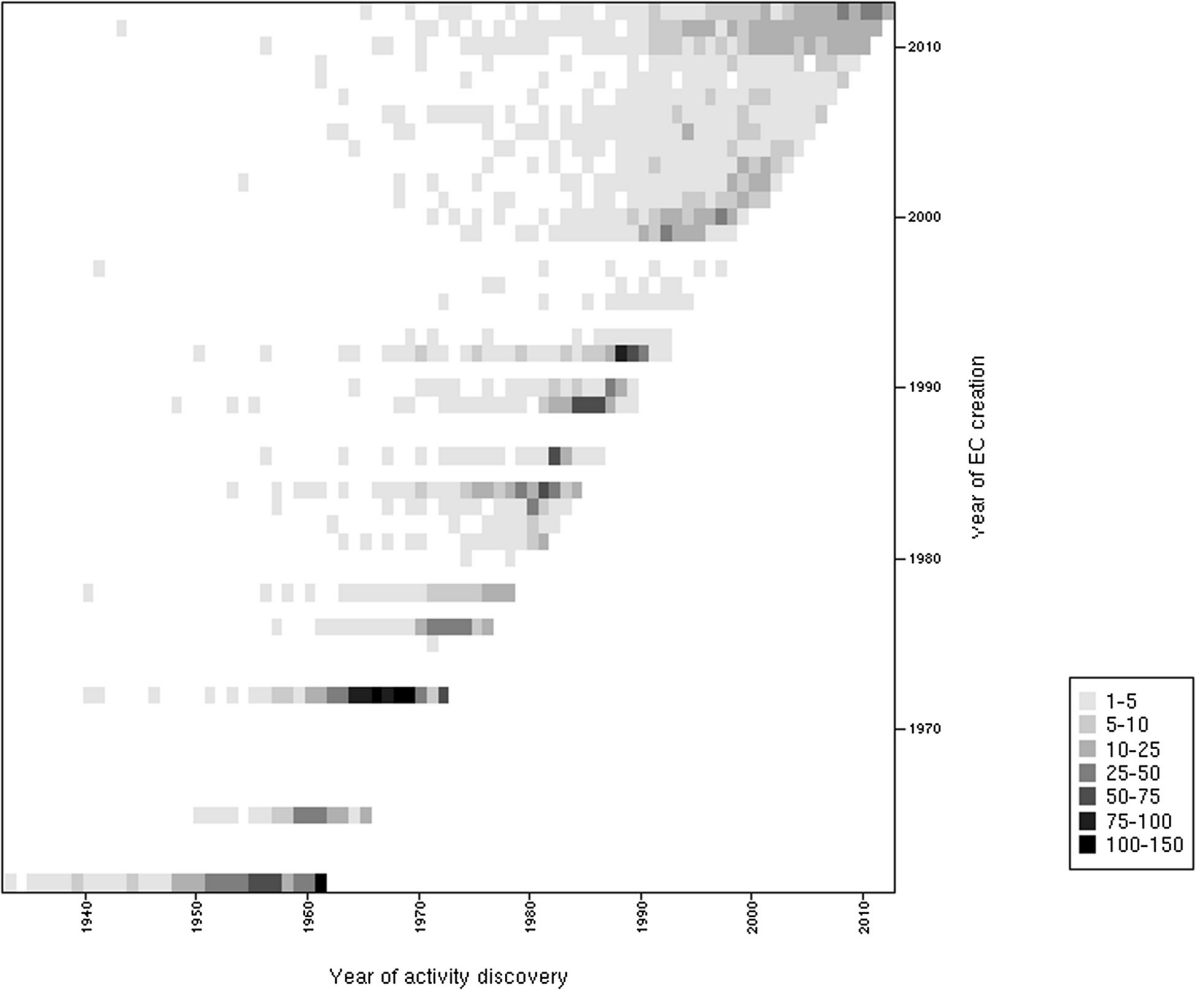
**Delayed knowledge in the EC classification.** Heatmap of the number of EC entries reported by the year of the activity discovery (X axis) versus the year of the corresponding EC entry creation (Y axis). The square’s shade of gray is proportional to the number of EC entries. A delay can be observed between the discovery of an activity and the creation of the corresponding EC number.

**Figure 4:**
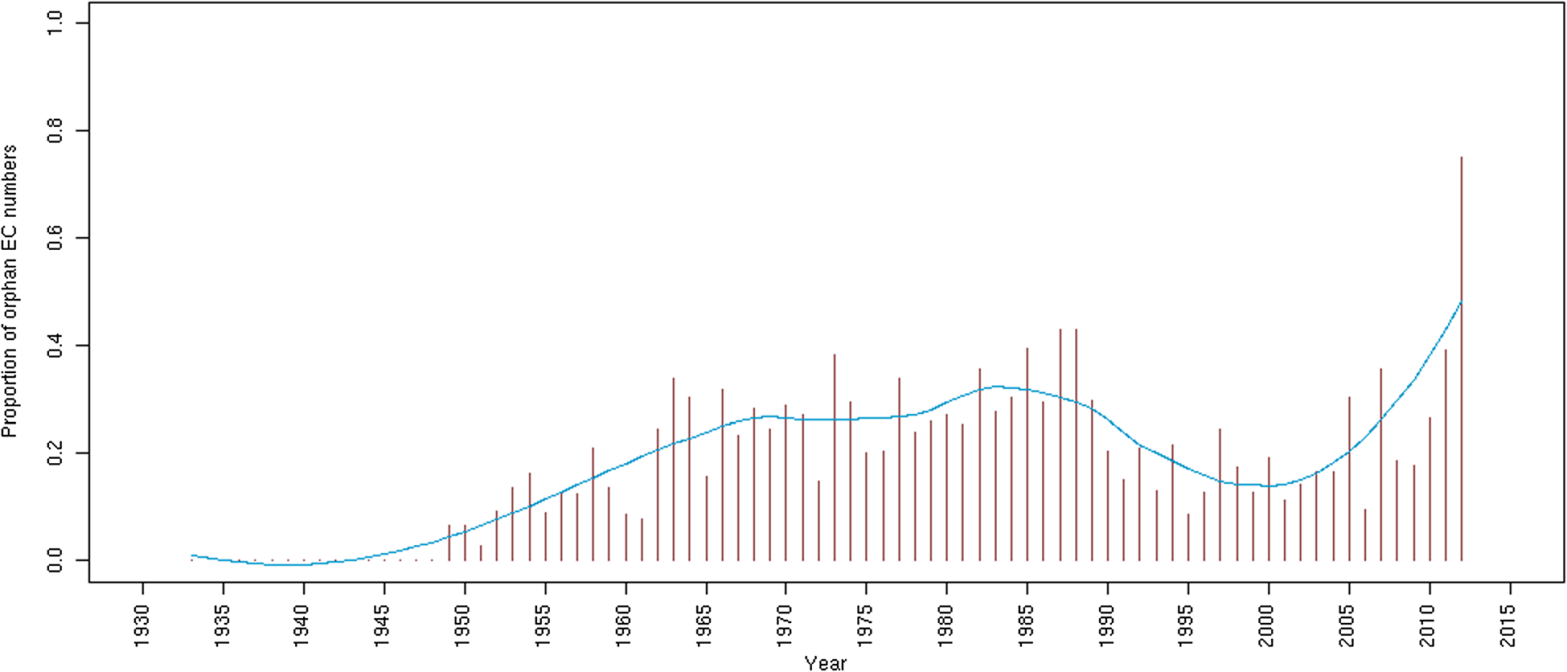
**Proportion of orphan EC activities by their year of discovery.** This bar plot represents the proportion of orphans among all discovered EC activities for a given year. In the aim to easily represent their evolution, the data is smoothed by a non-parametric local regression (blue line).

In comparison with the first study made by Peter Karp in 2003 [8], we observe a significant decrease in the number of orphan activities (−294 EC entries) while the number of EC entries has increased considerably (+1,360 entries) in the last ten years. To interpret this result, we performed a survey of the EC classification dynamics in terms of entry creations and updates (Figure 2). Since 2010, more than 800 EC numbers have been created and a substantial number of old entries have been re-classified (*i.e.* deleted or transferred to another entry). Over the last few years, the EC commission has considerably enhanced its activity and increased the coverage of the EC classification in terms of number of new enzymatic activities. Before the year 2000, the EC classification was not updated regularly each year, whereas new EC numbers are now created several times a year, suggesting that the Enzyme Commission tries to minimize the time between the publication of a new activity and its EC attribution. Nevertheless, many of these new EC entries correspond to older biochemical characterizations as depicted in Figure 3, where the delay between activity discoveries and corresponding EC creations is shown. This pitfall limits the search of enzymes in public databases since EC numbers are the only standardized way for scientists to publish an enzymatic activity associated with a protein sequence. Moreover, many recently characterized activities have no associated protein entries, see Figure 4. We can suppose that the annotations of the corresponding proteins were not updated accordingly with the correct complete EC numbers. This delay of knowledge in databases, which was reported by Yannick Pouliot and Peter Karp in 2007 [15], remains the case today and it impacts the evaluation of orphan enzymes because numbers of recently discovered enzymes are wrongly considered as orphans. These authors defined a strategy in order to determine which orphans might be salvageable and extrapolated that around 18% of them can be solved with a literature search. At the time of writing, this type of analysis was applied to a wide list of orphan EC numbers [16]. The authors found protein sequences for about 270 activities among 1,122 putative orphan enzymes that were extracted from databanks in 2009. Using their results and the current knowledge in databanks, protein entries for 112 false orphans could be updated with the corresponding activities and literature evidences.

**Figure 5:**
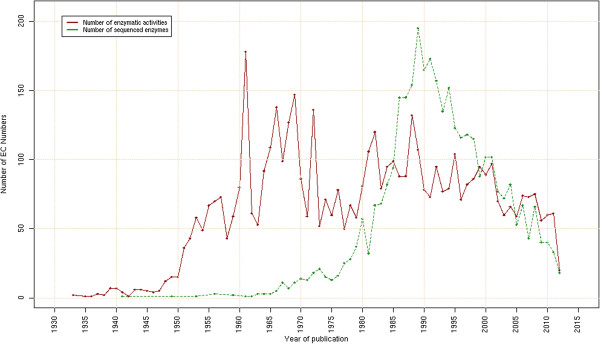
**The dynamics of enzyme discovery.** The solid red line represents the number of enzymatic activities by their year of discovery, which is estimated by using the earliest publication linked to the corresponding EC entries in IntEnz database. If no publication is mentioned, the year of creation of the EC entry is used instead. The dotted green line represents the number of activities associated to a biological sequence for the first time. The year of protein-EC number association is estimated using UniProt’s PubMed cross-references and by selecting only articles with less than ten other cited proteins in order to avoid publications related to the sequencing of large genomic regions. The artefact peak in 1961 is due to large number of created entries during the first EC meeting, where many activities were assigned to an EC number without any tractable publication.

To get a better view of the dynamics of the enzyme discovery in the past century, we computed the number of characterized activities over the years, represented by the solid red curve in Figure 5. As previously reported by Chen *et al.*[12] several phases can be observed. The 1930s and 1940s correspond to the beginning of biochemistry with a few numbers of characterized enzymatic activities. The 1950s and 1960s then saw an explosion of newly discovered activities due to technical progress in biochemistry and scientists’ increasing interest in this new field. This golden age of biochemistry took place in parallel with the progress in DNA knowledge and the emergence of molecular biology. These two complementary disciplines synergized in the 1980s and 1990s as shown by a second peak of enzymatic activities in Figure 5. Simultaneously, the number of activities associated for the first time with a protein sequence increased considerably (dashed green curve in Figure 5). Before this period, the purification and the direct sequencing of proteins were laborious and very few enzyme sequences were determined as it required highly purified polypeptides and was limited to short polypeptides. The improvements in molecular biology techniques, like DNA sequencing and expression cloning, permitted quick association between nucleic sequences (*i.e.* genes) and enzymes, whether the latter was long-known or recently discovered. The emergence of whole-genome sequencing projects and then, the Next Generation Sequencing (NGS) technologies should have eased the discovery of associations between genes and enzymatic activities. Unfortunately, since the year 2000 the number of newly discovered activities is not maintained at the established level and starts to dramatically decrease (Figure 5). It may be due to difficulties in publishing such biochemical characterizations, and also to the fact that funding is now directed towards other priorities. The gap between the number of sequences present in public databases and the number of characterized enzymes continues to increase dramatically [17-19]. In 2010, Hanson *et al.* pointed out the dual problem of increasing number of proteins of unknown function produced by genome projects, facing the orphan enzymes missing sequence information [20]. They suggested using experimental data and comparative genomics in order to predict candidate genes.

### Orphan enzymes in the metabolic world

It is important to distinguish the terms “enzyme” and “enzymatic activity”. The first designates a protein able to catalyze a chemical reaction and the second one the chemical reaction catalyzed by the enzyme. Therefore, an EC number does not represent the enzyme itself, but only the activity. As a consequence, non-homologous isoenzymes (*i.e.* with different ancestral origin) may share the same EC number as they catalyze the same enzymatic reaction. In the case of substrate promiscuity, different EC numbers may exist to give precision to the nature of transformed compounds. Otherwise, only one EC number may be available and represents a generic transformation that could occur on different substrates (*e.g.* alcohol dehydrogenase, hexokinase). The promiscuity aspect of enzymes is extensively described below. Besides, a same chemical transformation may be represented by different EC numbers when, for example, different cofactors are used. This multiplicity between related activities and EC numbers may lead to discrepancies in databases and mask some orphan enzymes. Another point, reported by Green *et al.*[21], is the ambiguity in the use of incomplete EC numbers that could lead to enzyme annotation errors in public databases. This is because incomplete EC numbers don’t distinguish between the lack of knowledge of the exact substrate specificity of an enzyme and the lack of an official EC number to describe the given activity. Consequently, the use of EC numbers may have introduced some biases in our survey. It should be noticed that the UniProt consortium is making improvements in the representation of the enzymatic activities through Rhea [22] and UniPathway [23] databases, which are focused on the definition of chemical reactions and metabolic pathways, respectively.

To complete our survey at the chemical reaction level, we performed a study on orphan enzymes using two metabolic databases, named KEGG (version 65.0) [24] and MetaCyc (version 17.0) [25]. The comparison of these two databases was extensively reviewed in a recent publication [26]. As a difference with EC nomenclature, KEGG and MetaCyc make a clear distinction between the chemical reactions and the enzymatic activities. MetaCyc has adopted a formal representation of the relation between proteins and chemical reactions they can catalyze and thus deals with the multiplicity of enzymatic activity-reaction relations. For example, if an enzyme is able to catalyze the same chemical transformation on a wide range of substrates (*i.e.* the substrate promiscuity of the enzyme), the different chemical reactions will be explicitly linked to the enzymatic activity description. In other cases, an EC entry may give only a general description of the overall reaction whereas the different steps of this chemical transformation may be more precisely described using several reaction steps. The results of our analysis are summarized in Table 1. About twice as many reactions are found in the two pathway databases in comparison to the ~5,000 EC entries. This high number of reactions is partly due to the multiple relations between enzymatic activities and reactions described above: in KEGG and MetaCyc, there is an average of 1.15 and 2.2 reactions per EC number, respectively. Conversely, a large proportion of these reactions correspond to enzymatic activities not described by a complete EC entry, reflecting the previously mentioned delay between an activity discovery and its official classification by the commission. In KEGG and MetaCyc, there are 4,588 and 4,497 reactions not linked to a complete EC number, respectively. As a consequence and as noted previously [12,27], the percentage of orphan enzymes may be underestimated using only the EC classification. It increases considerably when the estimation is made at the reaction level using metabolic resources: in KEGG and MetaCyc, 48% and 39% of the reactions are lacking associated protein or nucleic sequences, respectively.

**Table 1:** Statistics on orphan reactions in KEGG and MetaCyc metabolic databases

	**MetaCyc**	**KEGG**
Total number of non-spontaneous reactions	10126	9148
Number of orphan reactions	3929	4348
Number of reactions in a pathway	6873	6271
Number of orphan reactions in a pathway	1833	1716
Number of orphan reactions having a non orphan pathway neighbour	915	1223
Number of pathways	2002	150
Average number of reactions per pathway	4	80
Number of pathways with only non orphan reactions	1264	19
Number of pathways with only orphan reactions	155	0

Enzymes are classically studied through metabolic pathways, which are groups of activities taking part in a same biological process. In this survey, we studied the orphan enzyme content and their connectivity at the pathway level. As described previously [26], there are several key differences between the way the databases represent the notion of a pathway: KEGG pathways are a kind of mosaic of similar pathways predicted in different species; in MetaCyc, the overall reactions in a pathway are supposed to occur in a defined group of species. Therefore, there are 12 times more pathways in MetaCyc than in KEGG, as MetaCyc attempts to provide distinct pathway variants for a given metabolic process (Table 1). An important fraction of pathways (87% in KEGG and 36% in MetaCyc) contains at least one orphan activity. There is no pathway in KEGG containing only orphan enzyme activities, whereas it is the case for about a quarter of the MetaCyc pathways. This is explained by the very large number of reactions in KEGG pathways in comparison to MetaCyc (80 on average per pathway versus 4). Considering pathways containing a mix of orphan and non-orphan activities in KEGG and MetaCyc, an average of 26.0% and 39.5% of the reactions per pathway corresponds to orphan enzymes, respectively (Table 1). These statistics show that an important proportion of pathways are still not completely resolved at the gene level, which limits *in silico* reconstructions of genome-scale metabolic models [28,29]. To cope with this problem, computational tools were developed to find candidate genes for these missing enzymes by using genome and metabolic context-based methods [30-32]. The concept of these methods and the illustration of integrated tools using genomic and post-genomic data to link gene and function have been reviewed recently [33]. Another illustration is presented through the MicroScope platform as a combination of CanOE and phylogenetic profile methods [32,34]. Actually, these *in silico* predictions have not raised a lot of orphan cases despite the sophistication of the methods and their relative independence from classical sequence based methods. As many orphan enzymes (1,223 reactions in KEGG and 915 in MetaCyc) have pathway neighbors that are orphans themselves, one difficulty is the definition of correct genomic contexts including candidate genes and known enzymes. Furthermore, there is some part of the metabolism with a lot of missing knowledge like glycan and lipid pathways. For example, a number of orphan enzymes still exist in ether lipid metabolism, even if some recent progresses were made [35].

### Local orphan enzymes

From a taxonomic point of view, we propose to make the distinction between global and local orphan enzymes. Orphan enzymes were previously defined as activities having no associated gene in any organism, which we called here global orphans. In addition, a local orphan is an experimentally observed activity in at least one organism of a given clade with only associated sequences in organisms from other clades [36,37]. To illustrate this concept at the superkingdom level, we present here the example of the EC number 4.1.1.12, the aspartate 4-decarboxylase, which catalyzes the transformation of an L-aspartate in an L-alanine by releasing a molecule of CO_2_. In UniProt, 327 bacterial proteins are annotated with this EC number, including two SwissProt entries, but no eukaryotic or archaeal sequences can be found. Nevertheless, the aspartate 4-decarboxylase activity has been characterized in various mammalians (*e.g.* rat, pig, chicken) [38], making the EC number 4.1.1.12 a local orphan activity in eukaryotes. For the Archaea, there is no associated sequence and no literature evidence of its presence in this superkingdom. Thus, the aspartate 4-decarboxylase activity could be considered as absent in the Archaea.

**Figure 6:**
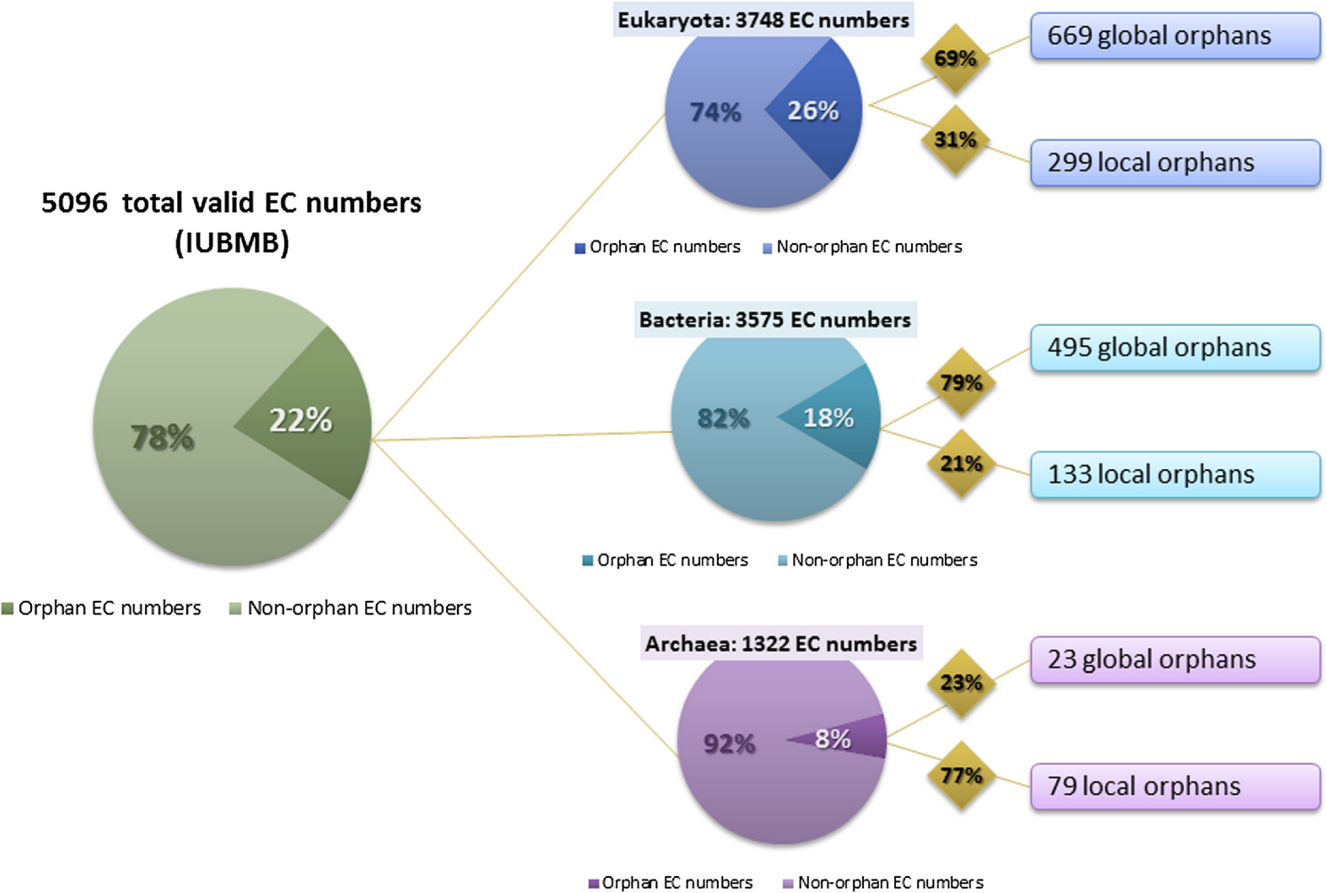
**Orphan and non-orphan EC number distribution across superkingdoms.** The green pie chart represents the proportion of orphan EC activities among all valid entries. Other pie charts represent the proportion of orphan activities among each superkingdom. An activity is considered as present in a superkingdom if at least one protein is annotated with corresponding EC number or the activity has been observed in an organism according to BRENDA database. The number and percentage of local and global orphans are given for each superkingdom. The small amount of characterized EC numbers in Archaea shows the obvious lack of knowledge about their metabolism.

To conduct a survey of local orphans, a resource of characterized activities in identified organisms is required and should be exhaustive enough to gather all the biochemical knowledge published in the past century. We used the BRaunschweig ENzyme DAtabase (BRENDA, version 2013), which is one of the major public resources on enzymes and enzymatic activities, and contains a very large spectrum of information related to them [39]. BRENDA is based on the EC number classification and gathers valuable information about biochemical experiments that were extracted from the literature. In complement to BRENDA that contains only manually annotated data, the FRENDA (Full Reference ENzyme DAta) and AMENDA (Automatic Mining of ENzyme DAta) subsections are based on an automatic text-mining of article abstracts and provide an exhaustive collection of organism-specific enzyme information. BRENDA was used in our survey to extract, for each enzymatic activity, a set of organisms for which the activity was observed. In combination with UniProt data, the proportion of global and local orphan enzymes at the superkingdom level was then estimated (Figure 6; the lists of local orphan and not observed EC numbers are available in Additional file 2: Tables S2.2 and S2.3 for Bacteria, S2.4 and S2.5 for Eukaryota, and, S2.6 and S2.7 for Archaea). Interestingly, we found that the proportion of orphan enzymes is higher in Eukaryota than in Bacteria (26% and 18%, respectively). Among the one thousand orphan activities in eukaryotes, a third corresponds to local orphans (31%) whereas the fraction is lower in Bacteria (21%). These slight differences could reflect a higher difficulty in experimental procedures to identify genes or proteins in eukaryotes. In Archaea, the low number of enzymatic activities (1,322 EC numbers), which are reported in BRENDA and UniProt, clearly illustrates our limited knowledge of metabolism of this superkingdom. In our study, the proportion of archaeal orphan enzymes is thus clearly underestimated. Indeed, new specific enzymatic activities need to be discovered as their chemistry shows many differences from other forms of life. Nevertheless, a high proportion of reported orphans in Archaea (77%) are local orphans, suggesting either homolog proteins could beeither homolog proteins could be candidates for these activities or specific isoenzymes have emerged during their evolution. A similar analysis was conducted by adding FRENDA/AMENDA data (Additional file 1: Figure S1.2). Surprisingly, the number of orphan enzymes considerably increased in each superkingdom with a high proportion of local orphans (52% for Eukaryota and Bacteria, and 91% for Archaea). These results should be taken with caution as FRENDA/AMENDA data is not subjected to manual curation (*e.g.* we found false-positive local orphans for Bacteria that correspond to heterologous expressions of eukaryotic proteins in *Escherichia coli* BL21). Nevertheless, this analysis demonstrates that, in addition to the 22.4% of global orphan, the proportion of EC numbers which are local orphans in at least one superkingdom is considerable and is estimated between 9.5% (BRENDA alone) and 33.5% (including FRENDA/AMENDA). Despite the observed decrease of orphans at a global level, this high number of enzyme activities (>30%), for which no or incomplete sequence information is available, remains problematic in our knowledge of metabolism.

Two reasons may explain this high proportion of local orphan enzymes. Firstly, non-homologous isofunctional enzymes, referred as NISE [40], may remain to be discovered. They correspond to proteins that evolved independently, but catalyze the same biochemical reactions. Therefore, these analogous enzymes cannot be detected by classical comparative genomics approaches, as they do not share any detectable sequence similarity. Secondly, candidate homologous proteins may exist for local orphans but remain to be experimentally confirmed and annotated in databanks. To address this second point, we conducted a preliminary analysis to find homologous proteins for all local orphan enzymes in a given superkingdom. For that purpose, we applied the PRIAM software (release of March 2013) [41] against all UniProt proteins from the Eukaryota, Bacteria and Archaea superkingdoms (see Additional file 1: Figure S1.3). PRIAM relies on a set of profiles (*i.e.* position-specific scoring matrices), which are supposed to be characteristic of protein modules sharing same enzyme activities (*i.e.* same EC numbers). We found that PRIAM is able to retrieve candidate proteins for a non-negligible fraction of local orphans previously defined using BRENDA data: 30% for Archaea and Bacteria, and 59% in Eukaryota (Table 2; the lists of candidate proteins for local orphan and not observed EC numbers are available in Additional file 3: Tables S3.1 and S3.2 for Bacteria, S3.3 and S3.4 for Eukaryota, and, S3.5 and S3.6 for Archaea). Even if these predictions cannot be transferred directly without supplementary bioinformatics analyses or experiments, they give strong clues on protein candidates for local orphan enzymes. Another interesting feature is the substantial number of putative candidates for activities that have never been seen in a given superkingdom (“not observed” columns in Table 2). Only 21% of not observed EC numbers in Archaea have candidate proteins whereas the total number of known enzymatic activities is low in this superkingdom (n = 1,322, Figure 6). This result is in agreement with the specificity of their metabolism, which may be a reservoir of new enzyme families and pathways. Conversely, the percentages of potentially resolvable local orphans and not observed enzymes in eukaryotes are higher than the two other superkingdoms, at 59% and 46% respectively. This suggests that the set of common enzymes between Bacteria and Eukaryota may be underappreciated in protein databanks and could be partially solved by a curation effort of eukaryotic genome annotations. As already illustrated, comparative genomics analyses between prokaryotes and eukaryotes are successful in finding common and specific enzymes in shared pathways [20]. These homology-based predictions of enzymatic functions could be also completed by probabilistic annotation of metabolic networks to increase the accuracy of this strategy [42].

**Table 2:** Potential candidates for local orphan enzymes retrieved by PRIAM

	** Archaea**		** Bacteria**		** Eukaryota**	
	**local orphan EC**	**not observed EC**	**local orphan EC**	**not observed EC**	**local orphan EC**	**not observed EC**
Total number	79	3774	133	1521	299	1348
Number of predictable	56	2247	115	817	150	718
Number of predicted	17	475	35	203	88	333
Percent of predicted	30%	21%	30%	25%	59%	46%
Number of candidate	400	9406	2929	11451	2996	9727

Not observed EC numbers correspond to entries than have never been associated to a protein or an organism in the superkingdom. Predictable EC numbers are entries having an associated PRIAM profile. A predicted EC number is an entry for which PRIAM detected a significant hit with at least one protein sequence (see Additional file 1: Figure S1.3).

### Enzyme promiscuity and protein families

Multifunctional enzymes are enzymes capable of playing several roles in metabolism by catalyzing different transformations that may occur in different pathways. Several kinds of multifunctionality can be observed. Some enzymes may show broad substrate specificity. This substrate promiscuity is a feature of enzymes able to catalyze the same chemical reaction on a variety of related compounds [43]. Other enzymes may catalyze different chemical transformations. One can observe proteins having two or more functional domains with different active sites [44]. The association of several domains within a protein, which is generally the result of a gene fusion event during evolution, may facilitate substrate conversion and regulation of the metabolic fluxes. Another origin of this catalytic promiscuity is the special case of moonlighting enzymes [45]. These proteins switch between activities under environmental changes according to their cellular localization, expression in a novel cell type, ligand or cofactor concentrations, oligomerization or complex formation with other proteins. A repository of multitasking proteins was recently set up and several examples of moonlighting enzymes may be explored [46].

The proportion of multifunctional enzymes may be underestimated [47,48] and only a few enzymes are described as multifunctional in databases: among the ~250,000 enzymes in Swiss-Prot, 5% are associated with two or more EC numbers and 3% with EC numbers having different classification at third-level. This proportion should dramatically increase when we will find a simpler way to detect them. Recently, a bioinformatic method based on reaction molecular signatures was proposed to predict catalytic and substrate promiscuity [49]. Using this method, a complementary study showed that highly promiscuous enzymes are more likely to be widespread in the tree of life [50]. Because multifunctional enzymes are so difficult to discover and annotate, they represent an interesting and relatively unexplored reservoir to find sequences for orphan enzymes. Quite often, biochemists discover a “new” activity performed by an enzyme known to catalyze other type of reactions [45]. The point is that the characterization of a novel protein generally leads to the discovery of only one function, but does not automatically include a search for all possible additional functions. Nevertheless, the characterization of supplementary *in vitro* activities does not necessarily imply the elucidation of *bona fide in vivo* functions.

**Figure 7:**
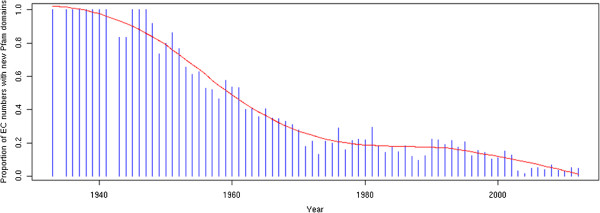
**Proportion of EC activities with new protein domains.** This bar plot represents the proportion of EC numbers having at least one new Pfam domain which was never associated to any enzyme before, by year of discovery. An EC number is considered to be associated to a new domain if this domain has never been seen associated to any other EC number discovered previously. Only EC numbers with at least one associated sequence were taken into account.

To explore the potential promiscuity of enzymes in a broader way, we conducted an analysis of enzyme activity/domain associations among all known enzymes using Pfam as a resource of domains [51]. We show that since the 1990s and despite the increasing number of available complete genomes in the last few years, the proportion of newly discovered activities associated to new domains (*i.e.* domains that were not previously associated to an enzyme) is continuously decreasing (Figure 7). Thus, the exploration of the functional diversity of known enzyme domains may be a good approach for finding proteins for new or orphan activities. Conversely, 22% of protein domains in Pfam remains without function and could be a reservoir of new enzyme families, considerably extending the enzyme world. A recent study successfully led to the discovery of new activities and pathways through the exploration of the enzymatic diversity of a protein family of unknown function [52]. On the structural side, a majority of enzyme activities are performed by a relative small number of protein superfamilies [53]. Indeed, we can observe an important diversity between the presence of a structural domain and the number of potential activities: using CATH as a resource of structural domains [54], there is an average of 6.37 EC numbers per CATH domain and of 27.20 CATH domains per EC class at third-digit. These observations reflect the importance of convergence in the evolution of enzymes [55]. In 2010, Omelchenko *et al.* found 185 enzyme activities with at least two structurally unrelated proteins [40]. The amount of NISE may even be revised upwards, as to our knowledge a systematic research of all potential structures performing the same activity has not been carried out. These complex relations between protein families and enzymatic activity diversity can introduce barely detectable, but easily spreadable, misannotations using homology based bioinformatics strategy during the annotation process [1]. Complementary analyses combining structural modeling, ligand docking and active site comparisons could lead to more accurate predictions and may open new ways to find candidate proteins for orphan enzymes.

## Conclusion

Despite an observed decrease of the number of orphan enzyme activities over the last ten years, the orphan enzyme challenge remains important: more than 30% of the enzymatic activities reported in the EC classification have no or incomplete sequence information. Though NGS, combined with improvements in sequence analysis methods, produces an exponential growth of genomic data, an explosion in the number of newly discovered activities has not occurred unlike the 80’s when the democratization of molecular biology techniques took place. This lack of knowledge is obviously problematic in the overall comprehension of metabolism and in potential biotechnological applications like biocatalysis.

As shown in our survey and as previously reported [20], a more systematic use of comparative genomics across superkingdoms may help to solve part of the local orphans. For the global ones, a delay of knowledge in databases still exists and could be resolved by intensive bibliographical searches. In this way, the Orphan Enzyme Project initiative [56] recently conducted a systematic analysis of databases and publications, and found protein sequences for about 270 presumed orphans among an initial list of 1,122 activities established in 2009 [16]. Similarly to what is done for protein structures with the PDB [57] and nucleic sequences by the INSDC (International Nucleotide Sequence Database Collaboration) [58], the design of a central and common scientific framework to submit enzymes with their activities is of priority to reduce the loss of knowledge between publications and databases. Indeed, collaborative initiatives were recently established to discover new activities and enzymes: the Enzyme Function Initiative [59] which addresses the challenge of assigning reliable functions to enzymes discovered in bacterial genome projects, and the COMBREX project [60], connecting computational and experimental biologists to improve protein annotation and proposing grants to experimentally validate new functions. These kinds of projects combining *in silico* and wet lab strategies should lead to a breakthrough in the discovery of new enzymes and activities since classical sequence based methods have lost momentum in function prediction. In fact, several recent studies have successfully applied this approach by exploiting mass-spectrometry or high throughput enzymatic assay experiments and computational methods using sequence similarity networks, genomic contexts, structural modeling with metabolite docking and active site comparison [52,61,62]. Another field of research concerns non-protein enzymes. The most well-known are ribozymes and all kinds of protein-RNA complexes, like ribosomes, that are a real challenge to study and extremely hard to discover [63,64]. The existence of active RNA has been known for a long time, but expertize in this area is far from being as exhaustive as in classical biochemistry. More recently, the discovery of a glycolipid playing a “membrane protein integrase” role in *Escherichia coli* has pushed back the limits of known catalytic activities [65]. After all, not only should we enlarge the limits of potential catalysts, but also enlarge the limits of the known metabolites. Progress in metabolomics will certainly catalyze the discovery of numerous chemical compounds orphan of activities.

## Reviewers’ comments

We thank the reviewers for their comments. We have revised the manuscript taking into account their remarks.

### Reviewer 1 (First Round): Dr. Michael Galperin

The paper by Sorokina *et al.* addresses an important question and includes some interesting results. However, I think that in order to justify publication in Biology Direct, the paper needs to be much better written. The current version is something intermediate between a review and a regular research paper and does not make for either a good review or a good research paper. As an example, I would suggest moving Figure 1 to Supplementary Materials (it is not a new result) and moving Figure S2 into the main text (it is a new result).

Authors’ response: *Our article is not a regular research article but a review paper written in a format similar to previous studies listed in Figure*1*. It includes updated analyses of existing data from public databanks that substantially enhance our knowledge about orphan enzymes. We thus decided not to move Figure*1*to Supplementary Materials as it resumes previous studies. Figure S2 (now S1.2) is an estimation of orphan enzymes at the superkingdom level based on non-curated data from FRENDA and AMENDA whereas Figure*6*was made using manually curated data. Therefore, we prefer not to move Figure S1.2 to the main text.*

In addition, I am afraid that the current version of the manuscript does not really benefit the scientific community as it simply enumerates the enzymes in each category without providing the specific lists of these enzymes. I could support publication of this paper only after the authors include (at least as Supplementary Materials) the lists of global and local orphans from Figure S2. Unless this is done, the data in Figures 2, 3 and 4 cannot be independently verified and the entire manuscript cannot be considered acceptable for publication.

Authors’ response: *We added the lists of global and local orphans and proteins in Supplementary Materials 2 and 3.*

Finally, the entire paper looks like a promotion for the Orphan Enzymes Project [http://www.orphanenzymes.org, ref. 49]. However, according to the Orphan Enzymes web site, this project is also the subject of an upcoming paper “Finding sequences for over 270 orphan enzymes” (currently in press). The reviewers should have been provided the text of that other paper to ensure that there was no significant overlap between the two.

Authors’ response: *We have no relation or contact with the Orphan Enzymes Project and had not access to their upcoming paper at the time of writing the present article. This article is now published and sentences were included in the main text to present their work.*

To help revision of this manuscript, I provide below some specific examples of the poorly formulated sentences. However, the entire text must be carefully revised and made less descriptive and more concise.

1. The Abstract needs to be revised to clearly explain what are the new results communicated in this work. Right now, the new results seem to start from “Besides, we extended our study”? Please rewrite the first 4 sentences of the Abstract to explain what exactly was the goal of this work and what exactly has been done.

2. The statement in the Abstract “We developed a simple strategy to rescue these local orphan enzymes” is totally enigmatic and has to be deleted or reformulated.

3. The last sentence of the Abstract does not seem relevant to the rest of the text. Please either delete or at least reformulate.

Authors’ response: *Part of the abstract has been rewritten according to the reviewer suggestions.*

4. The Introduction could (and should) be made more compact and succinct. That said, the last paragraph of the Introduction contains a much better description of the work presented in this paper than the Abstract does.

Authors’ response: *We removed the definition of the EC nomenclature but we think that it is important to keep a description of previous analysis reviews on orphan enzymes in the introduction.*

5. Citations of the enzyme and EC number databases in the Introduction and other sections of the paper present are unfortunately biased. The authors should, at the very least acknowledge the official web sites of the EC classification, the IUBMB list (http://www.chem.qmul.ac.uk/iubmb/enzyme/) and/or the ExplorEnz (http://www.enzyme-database.org, PMID: 18776214) as well as the ENZYME database (http://www.expasy.org/enzyme/ PMID: 10592255), That would also make it unnecessary to explain the organization of the EC system in the Introduction section. INSDC should be cited (PMID: 23180798). The section on Enzyme promiscuity should probably mention the availability of the MultiTaskDB (http://wallace.uab.es/multitask/, PMID: 24253302).

Authors’ response: *Suggested references have been added.*

### Reviewer 2 (First Round): Dr. Daniel Haft

The manuscript submission by Sorokina *et al.*, “Profiling the Orphan Enzymes”, functions fairly well as a review article on the chronology of the growth of EC numbers with and without associations with specific sequences. The authors define a problem space - identifying enzymes that have no representative in some superkingdom -. They introduce a strategy for generating lists of candidate sequences to fill the void. The revised form of the manuscript now provides lists of these candidate sequences in supplementary materials, rather than their count only, and it clearly warns that the associations offered by their technique are in no way validated.

The strategy relies on PRIAM, an update from March 2013. But there is no discussion of how PRIAM itself is formed and whether its design could be appropriate to the task. PRIAM was described in 2003, and relies on MKDOM. Therefore, PRIAM requires an unsupervised domain definition algorithm to find signature regions one enzyme has but another enzyme lacks. The domain could be a C-terminal extension with no relevance to enzyme function, and could be eukaryotic only, but PRIAM would make it a signature. Should this method be used to identify probable “local orphan enzymes” in the archaea? Not without validation.

Other homology strategies might do as well PRIAM or better, such as searching for bi-directional best BLAST hit matches that link a known exemplar of enzyme function in one superkingdom to a homolog in another superkingdom. The PRIAM strategy itself could have been benchmarked somewhat be seeing how much its predictions vary from one version to the next. Readers are strongly cautioned that the output from the PRIAM strategy should be viewed only as anecdotal evidence, appropriate to a review article, that simple homology methods could generate lists of sequences that contain candidates to represent the first extension into a new superkingdom of enzymatic activities that have been assigned to sequences in other superkingdoms.

Authors’ response: *This strategy is not a methodological development but just a way to estimate if candidate proteins for local orphans could be retrieved by homology search. We agree that PRIAM profiles have limitations but, as far as we know, it is one of the best tools to track potential conserved domains which are enzyme specific and have a wide coverage of Swiss-Prot enzymes. BBH cannot be computed for all the Swiss-Prot enzymes as many of them are not from complete organisms. As mentioned in the manuscript: “these [PRIAM] predictions cannot be transferred directly without supplementary bioinformatics analyses or experiments”.*

As a review, the manuscript did not do justice to the methods that might be used to find orphan enzymes in general, or domain orphans. In particular, Yamada *et al.* (ref 27) struck me as a landmark demonstration of data mining combined with comparative genomics for finding complete sequence orphans. The method would work even better for superkingdom orphans. Because that work followed predictions with validations, it represents a standard that should be discussed in any review article on matching sequences to orphan EC numbers.

Authors’ response: *We introduce the main methods of finding candidate genes for global or local orphans and some of their limitations. But, we do not wish to develop more deeply these methods for three reasons: (1) a complete review of these methods would require a dedicated article (2) a methodological review should be done by a third party since authors of the paper are involved in methodological developments on this topic (i.e. the CANOE method was published the same year as Yamada et al. paper) (3) a review has recently been published and presents a practical description of these methods (El Yacoubi et al. 2014, a reference to this paper was added in our article). For information, the two experimentally tested enzymes in Yamada et al. are not supported by enough evidence to validate that they are good candidates for the two orphan activities: (1) the two tested activities are amino acid transaminases, which are known to have in vitro substrate promiscuity (2) the candidate protein (UniProt AC Q8R5Q4) for the histidine transaminase activity has a TIGRFAM result corresponding to HisC protein (TIGR01141), which catalyzes the transamination of imidazole acetol-phosphate in the context of the histidine biosynthesis. Furthermore, the corresponding gene (TTE2137) is in the hisGDCBHAFI operon confirming that this protein should be involved in the histidine biosynthesis and not in the degradation process via the histidine transaminase activity. (3) the candidate protein (UniProt AC Q8DTM1) shares more than 50% of amino acid identity with biochemically characterized aspartate aminotransferases (UniProt ACs P23034, Q59228). This activity is more coherent with the asparaginyl tRNA synthetase genomic context than the asparagine aminotransferase activity proposed by Yamada et al., an activity described only in eukaryotes for asparagine degradation. These two cases are really good examples to illustrate the difficulty in interpreting in vitro activities to elucidate bona fide in vivo functions.*

The work introduces a workflow for using PRIAM to find sequences that might resolve numbers of local enzyme orphans. The lack of any testing of the workflow’s results or consideration of whether PRIAM’s design makes it a good choice was a problem. The revision, including author responses to the reviews, helps cement that this work serves as a review article only, and no tested new method is presented. Even in the revised form, the discussion of the PRIAM workflow is a bit troubling. Does the article title, “Profiling the Orphan Enzymes”, refer to PRIAM profiles as used in the untested workflow? If so, a revised title might be more appropriate.

Authors’ response: *The title is not related to PRIAM profiles. The aim of our review is to analyze and discuss the orphan enzyme problem in the light of the current knowledge in public databanks.*

### Reviewer 3 (First Round): Dr.Daniel Kahn

This reviewer provided no comments for publication.

### Reviewer 1 (Second Round): Dr. Michael Galperin

Previous authors’ response: *We added the lists of global and local orphans and proteins in Supplementary Materials 2 and 3.*

These lists could be very useful for future studies. My only concern is with the confusing terminology used to name the enzyme groups. The authors use the term “missing enzymes” for the enzymes that are absent (not encoded), rather than missing (not found), in the given taxonomic group. Instead, they use the term “local orphans” for the enzymes that everybody else in the world refers to as “missing enzymes”.

1. Enzymes (EC numbers) that are not associated with any sequences are referred to as “global orphans” even though many (probably most) of these enzymes have been described in a single species, or a group of closely related species, and therefore represent “lineage-specific orphans”, rather than “global orphans”. It would be helpful to explain this in the text to avoid confusion.

Authors’ response: *For the definitions of global and local orphans, we use the same as the ones of Orth et al. 2010. These definitions are given in the main text. For global orphans, it is very difficult to estimate if they are mostly associated to specific lineages as experimental data is limited and is far from covering the metabolic diversity of living organisms.*

2. Enzymes (EC numbers) that have not been reported in bacteria are referred to in Table S2.3 as “Missing enzymes in Bacteria”. In all previously published literature, “missing enzymes” referred to the enzymatic activities that are expected - or known - to be present in at least some bacteria but have not yet been assigned to any sequence. Thus, “Missing enzymes in Bacteria” are the ones that have been reported in certain eukaryotes and are not even expected to be encoded in any bacteria. As a result, there are 1521 enzymes “missing in Bacteria” and 3773 enzymes “missing in Archaea”. Again, if the authors choose to keep this - unconventional and counterintuitive - group name, they should explain it in the text to avoid confusion.

Authors’ response: *We agree with the reviewer that the term “missing” is confusing. We have replaced “missing” by “not observed” in the additional files and in the main text.*

Although the text has been significantly improved, I remain puzzled by the expression “Rescuing the local orphans”. What do the authors mean by “rescuing” here, probably not something that is covered by the existing dictionaries?

Authors’ response: *The term “rescuing” has been removed.*

### Reviewer 2 (Second Round): Dr. Daniel Haft

The revised form of the article makes it clearer that it is a review, not original research, and that a method they introduce produces only a suggestive view, not scientifically validated results. But it is still a little troubling. The title seems to speak of the new method, and there is no peer-reviewed endorsement of that method her.

Authors’ response: *These points are discussed in the first round of the review.*

## Competing interests

The authors declare that they have no competing interests.

## Authors’ contributions

OL and DV supervised the project. CM contributed to the design of the study and to finalize the manuscript. MSo performed the statistical analyses and the data gathering. MS made the PRIAM analysis. MSo, MS and DV wrote the manuscript. All authors read and approved the final manuscript.

## Supplementary Material

Additional file 1: Figure S1.1 Orphan enzymatic activity distribution across the EC classification **Figure S1.2.** Orphan and non-orphan EC number distribution across superkingdoms including data from BRENDA, FRENDA and AMENDA. **Figure S1.3.** Strategy for local orphan enzyme rescuing using PRIAM. Click here for file

Additional file 2 List of global and local orphan enzymes. Click here for file

Additional file 3 List of retrieved sequences through the PRIAM search. Click here for file
