# Supplementary material for: Profiling the orphan enzymes
Source: Biol Direct. 2014 Jun 6;9:10. doi: 10.1186/1745-6150-9-10 (PMC4084501; doi:10.1186/1745-6150-9-10)

Additional file 1

Profiling the orphan enzymes

Maria Sorokina, Mark Stam, Claudine Médigue, Olivier Lespinet and David Vallenet

Figure S1.1. Orphan enzymatic activity distribution across the EC classification

The EC classification is represented in a tree hierarchy. The branch length is proportional to the orphan's rate in the given class or subclass. Orphan proportions are mentioned for the 6 main EC classes.

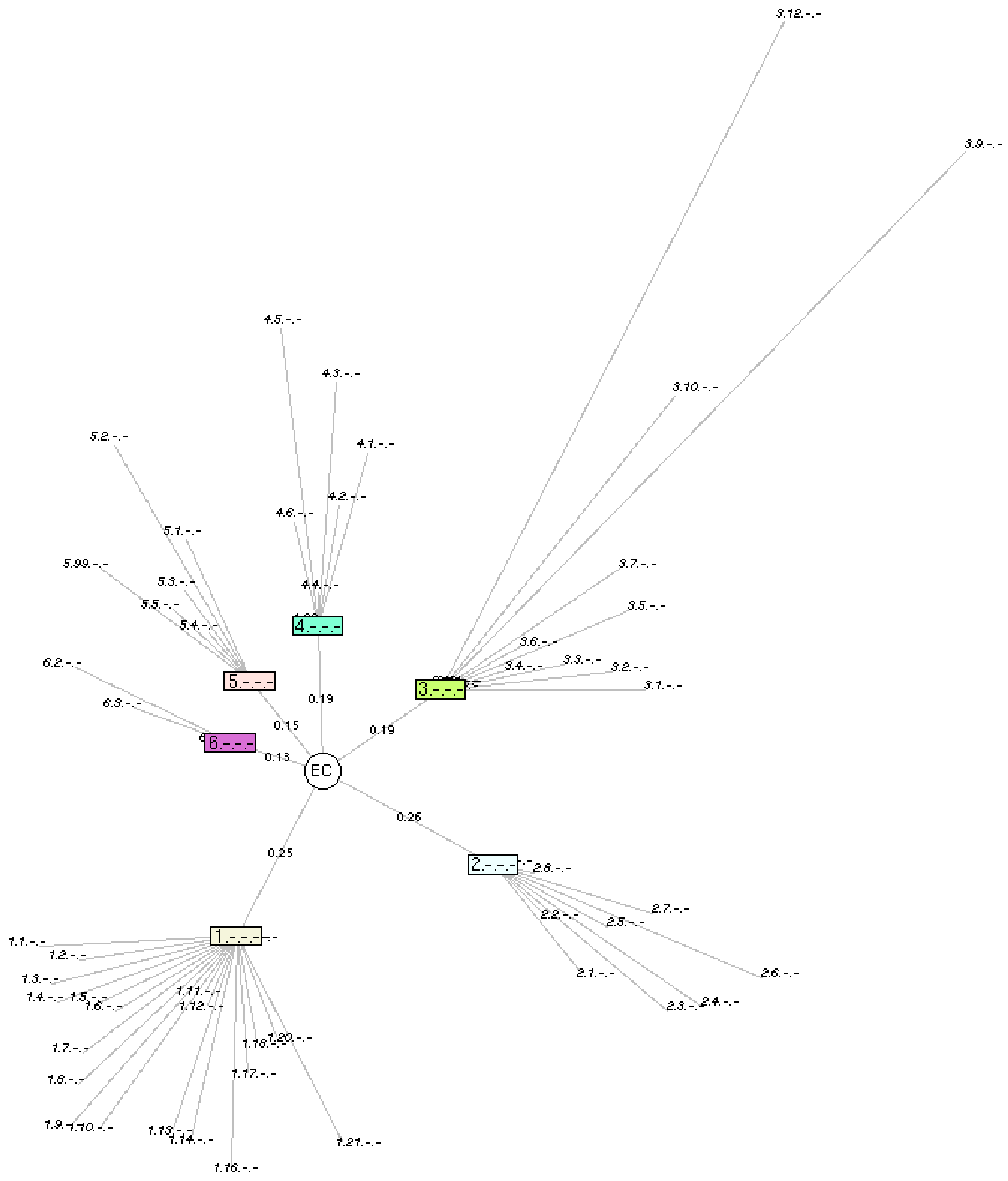

Figure S1.2. Orphan and non-orphan EC number distribution across superkingdoms including data from BRENDA, FRENDA and AMENDA

The green pie chart represents the proportion of orphan EC activities among all valid entries. Other pie charts represent the proportion of orphan activities among each superkingdom. An activity is considered as present in a superkingdom if at least one protein is annotated with corresponding EC number or the activity has been observed in an organism according to BRENDA, FRENDA and AMENDA databases. The number and percentage of local and global orphans are given for each superkingdom. Considering the textmining results provided by FRENDA and AMENDA, a larger amount of EC numbers could be linked to each superkingdom in comparison to BRENDA alone. Nevertheless, information contained in FRENDA and AMENDA should be considered with caution, even if textmining methodology improves ceaselessly.

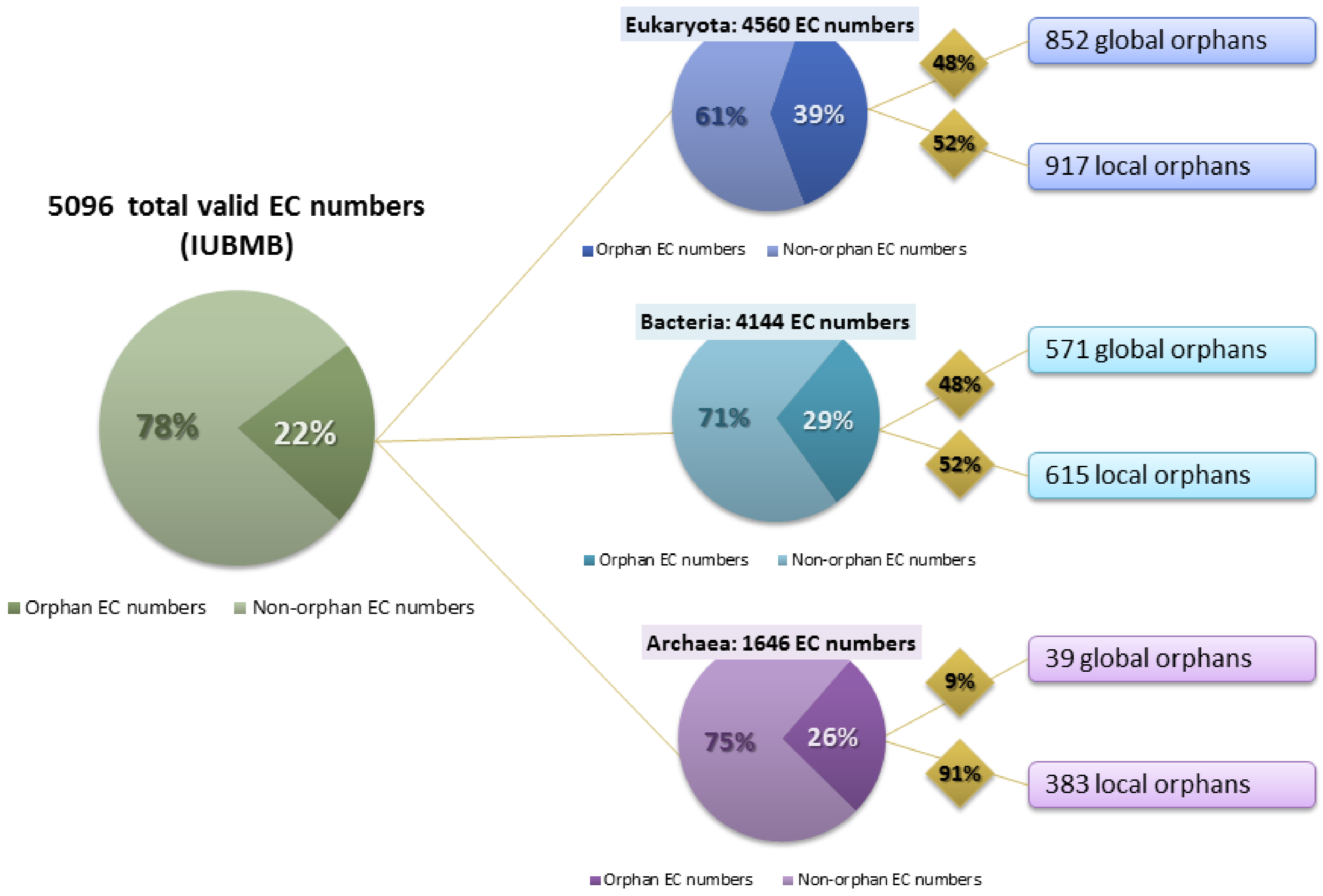

Figure S1.3. Strategy for local orphan enzyme rescuing using PRIAM

We started with two EC number lists for each superkingdom. The first one is the list of local orphan enzymes derived from BRENDA; the second one is the list of EC numbers not observed in the superkingdom. We used a two-step strategy to rescue local orphan enzymes using PRIAM. Firstly, we divided UniProt into three subsets (one for each superkingdom) and we screened each list (i.e. orphans and not observed EC numbers) with corresponding PRIAM profiles using PSI-BLAST. We obtained sets of putative candidates with predictable activities. Secondly, we used the PRIAM_search utility against the putative candidates keeping "the best overlap" hits with an e-value <= 1e-5. It allowed us to obtain sets of candidate sequences for local orphans and not observed activities.

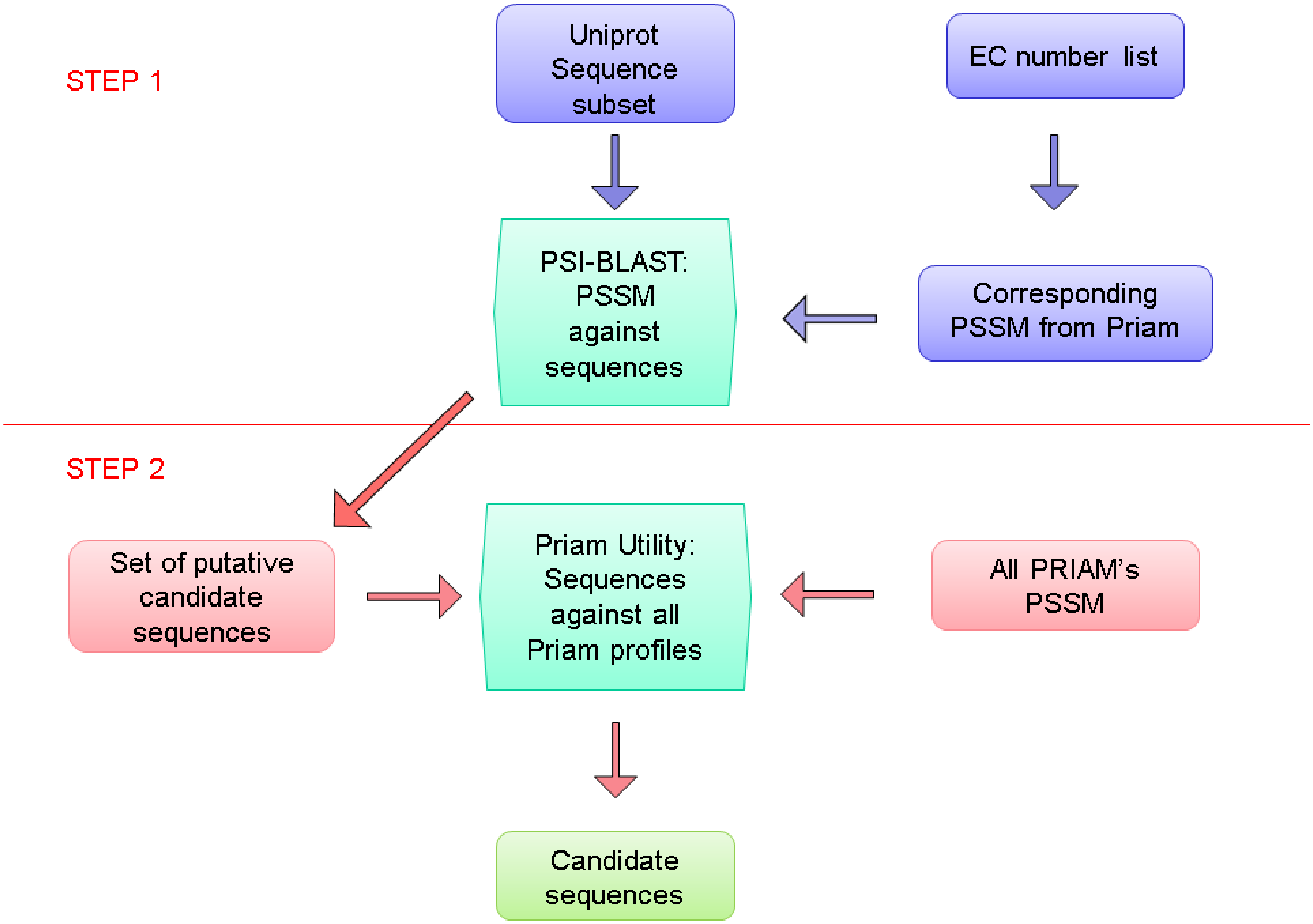

Supplement: Additional file 1: Figure S1.1 — Orphan enzymatic activity distribution across the EC classification Figure S1.2. Orphan and non-orphan EC number distribution across superkingdoms including data from BRENDA, FRENDA and AMENDA. Figure S1.3. Strategy for local orphan enzyme rescuing using PRIAM. [file 1745-6150-9-10-S1.pdf]
